# Mechanisms, combination therapy, and biomarkers in cancer immunotherapy resistance

**DOI:** 10.1186/s12964-024-01711-w

**Published:** 2024-06-19

**Authors:** Manshi Yang, Mengying Cui, Yang Sun, Shui Liu, Weibo Jiang

**Affiliations:** 1 Department of Hepatobiliary and Pancreatic Surgery, The Second Hospital of Jilin University, Changchun, 130041 China; 2 Department of Orthopaedic, The Second Hospital of Jilin University, Changchun, 130041 China

**Keywords:** Cancer, Immunotherapy resistance, Mechanism, Combination therapy, Biomarker

## Abstract

Anti-programmed death 1/programmed death ligand 1 (anti-PD-1/PD-L1) antibodies exert significant antitumor effects by overcoming tumor cell immune evasion and reversing T-cell exhaustion. However, the emergence of drug resistance causes most patients to respond poorly to these immune checkpoint inhibitors (ICIs). Studies have shown that insufficient T-cell infiltration, lack of PD-1 expression, deficient interferon signaling, loss of tumor antigen presentation, and abnormal lipid metabolism are all considered to be closely associated with immunotherapy resistance. To address drug resistance in tumor immunotherapy, a lot of research has concentrated on developing combination therapy strategies. Currently, ICIs such as anti-PD-1 /PD-L1 antibody combined with chemotherapy and targeted therapy have been approved for clinical treatment. In this review, we analyze the mechanisms of resistance to anti-PD-1/PD-L1 therapy in terms of the tumor microenvironment, gut microbiota, epigenetic regulation, and co-inhibitory immune checkpoint receptors. We also discuss various promising combination therapeutic strategies to address resistance to anti-PD-1/PD-L1 drugs, including combining these therapies with traditional Chinese medicine, non-coding RNAs, targeted therapy, other ICIs, and personalized cancer vaccines. Moreover, we focus on biomarkers that predict resistance to anti-PD-1/PD-L1 therapy as well as combination therapy efficacy. Finally, we suggest ways to further expand the application of immunotherapy through personalized combination strategies using biomarker systems.

## Introduction

With its approval in 2011, the anti-cytotoxic T lymphocyte antigen 4 (CTLA-4) antibody ipilimumab started a new era of cancer treatment, as immune checkpoint inhibitors (ICIs) that primarily block programmed death 1 (PD-1) as well as CTLA-4 became the cornerstone of immunotherapy [1]. To date, eight drugs targeting the PD-1/programmed death ligand 1 (PD-L1) axis have been approved by the US Food and Drug Administration (FDA) for cancer treatment [2, 3]. However, while ICI use has expanded therapeutic options for many cancer types, it has failed to live up to expectations. Nearly 70% of patients achieve only transient T-cell recovery and no sustained benefit during anti-PD therapy [4].

ICI resistance is the main reason for poor efficacy. Tumor-associated macrophages (TAMs), myeloid-derived suppressor cells (MDSCs), and regulatory T cells (Tregs) in the immune microenvironment exert a negative effect on antitumor therapy, forming an immunosuppressive microenvironment that leads to ICI resistance [5]. In addition, antibiotic-mediated dysbiosis of the gut microbiota has been associated with decreased anti-PD therapeutic efficacy. The composition and abundance of gut microbiota may modify the effects of ICIs [6]. Similarly, epigenetics is also relevant to the establishment of drug resistance. For example, the epigenetic stability of exhausted T cells affects sustained immune activation mediated by anti-PD therapy [7]. Activation of other immune checkpoints, such as T-cell immunoglobulin mucin 3 (TIM-3), lymphocyte activation gene-3 (LAG-3), and V-domain Ig suppressor of T-cell activation (VISTA), can mediate T-cell exhaustion independently of the PD-1/PD-L1 pathway, inducing drug resistance [8–10]. Indeed, numerous resistance mechanisms prevent anti-PD therapy from activating an effective immune response. PD-1 deficiency directly affects the therapeutic efficacy of PD-1/PD-L1 inhibitors [11]. Inadequate T-cell infiltration, presumably due to active expression of pro-angiogenic factors such as vascular endothelial growth factor (VEGF) or the absence of phosphatase and tensin homolog (PTEN), is an important reason for anti-PD therapy failure [12, 13].

Combination therapy, which is considered the most promising cancer immunotherapy strategy, has had success in overcoming partial drug resistance. A classical immunotherapy combination of a PD-1 inhibitor and a CTLA-4 inhibitor has been approved to treat a wide range of cancers, such as mesothelioma and non-small cell lung cancer (NSCLC), suggesting that dual or even multiple blockade of immune checkpoints have the potential to resolve the resistance mediated by the increased variety of checkpoints [14, 15]. One approach to address the immunosuppressive microenvironment is to deplete immunosuppressive cells and reshape the tumor microenvironment (TME). Entinostat, which reduces MDSCs, and paclitaxel, which reprograms TAMs, can help shape a suitable TME for anti-PD therapy [16, 17]. Moreover, the combination of targeted therapy with anti-PD therapy addresses multiple resistance pathways. For example, epidermal growth factor receptor tyrosine kinase inhibitors (EGFR TKIs) regulate the TME and promote antigen presentation; in combination with PD-1 inhibitors, they activate potent antitumor immunity [18, 19]. Recently, it was confirmed that TYRO3 inhibits anti-PD therapy-mediated ferroptosis and promotes the formation of an immunosuppressive TME, which is of great significance in inducing drug resistance. However, the clinical efficacy and risks of combining TYRO3 inhibitors with anti-PD-1 antibodies remain unknown [20].

Biomarkers are crucial for the implementation of combination therapy. Selecting patients who will respond to anti-PD-1/PD-L1 treatment should improve clinical outcomes for those patients and reduce the economic burden associated with treatment [21]. Patients testing positive for PD-L1 were thought to be responsive to anti-PD therapy; however, a subset of patients with PD-L1-negative squamous NSCLC respond to anti-PD therapy [22]. In patients with hepatocellular carcinoma (HCC), serum PD-1 levels are associated with a favorable overall survival (OS), while serum PD-L1 predicts a poor prognosis [23]. Different forms of PD-1/PD-L1 vary in significance in each tumor type. Therefore, establishing a comprehensive biomarker system will help to select patients with a better prognosis, evaluate the risks associated with combination therapy, and lay the foundation for precision medicine.

In this review, we summarize the mechanisms of immunotherapy resistance, including the TME, gut microbiota, epigenetic regulation, and co-inhibitory receptors of immune checkpoints. We also discuss the synergistic effects of combination therapeutic strategies, and examine biomarkers and solutions for drug resistance in immunotherapy, with the aim of guiding patient selection and prognostic evaluation, and improving efficacy and clinical response.

## Drug resistance mechanisms of immune-related events with immunotherapy

### The TME

**Fig. 1:**
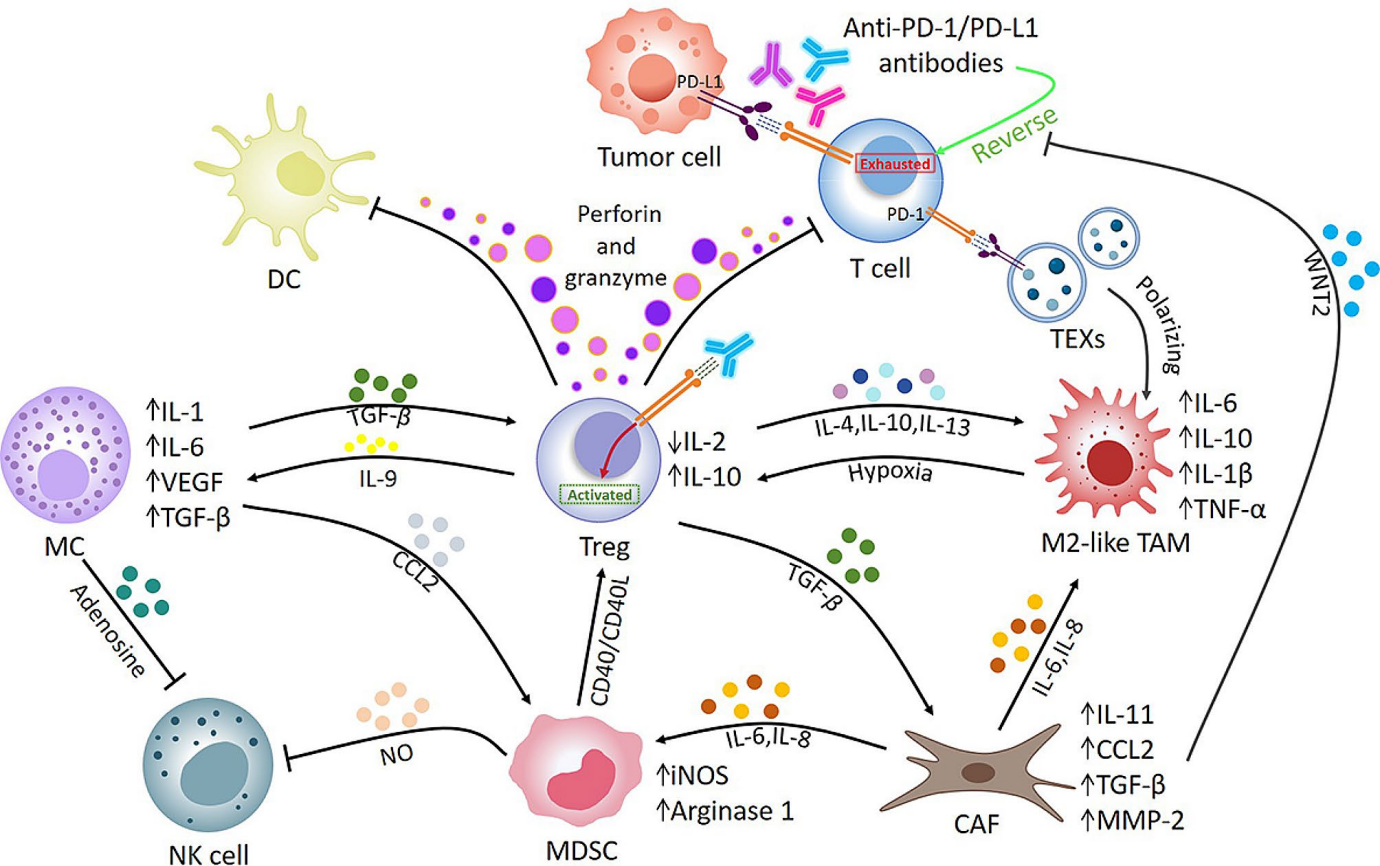
Establishment of immunosuppressive microenvironment. Immunosuppressive networks pro-tumor cells formation in the TME. Immunosuppressive cells impair anti-tumour immunity induced by ICIs through multiple pathways

As the key ecological habitat for tumors, the TME has a key role in the balance between antitumor immunity and tumor evasion. In addition to the “good guys” responsible for antitumor immunity, such as cytotoxic T lymphocytes (CTLs), natural killer (NK) cells, and dendritic cells (DCs), many “bad guys” are driving immunosuppression in the TME [24]. M2-like TAMs, MDSCs, and Tregs, as well as transforming growth factor-β (TGF-β) and interleukin (IL)-10 exert varying degrees of inhibitory effects at various stages of antitumor immune activation [24]. As a result of these negative feedback regulators of anticancer immunity, immunotherapies centered on immune cell activation, such as anti-PD-1 antibodies, frequently have limited efficacy [5]. Therefore, targeting and inhibiting the “bad guys” in the TME may be a new, useful strategy to rescue the efficacy of ICIs (Fig. 1).

#### TAMs

Two TAM phenotypes have distinct roles in the TME. Because TAMs are highly plastic TME components, exposure to interferon-gamma (IFN-γ) or lipopolysaccharide molecules shifts them toward an M1 phenotype. However, TAMs exhibit an M2 phenotype in the presence of IL-4, IL-13, and IL-10. M1-like TAMs present antigens and express pro-inflammatory cytokines such as IL-12 and tumor necrosis factor-alpha, helping to activate the immune response and eliminate tumor cells. In contrast, by secreting IL-10 and TGF-β, M2-like TAMs control inflammation and exhibit immunosuppressive functions [25]. M2-like TAMs are frequently found in tumors, where they interact with tumor cells thereby suppressing tumor immunity and promoting tumor development. By changing their phenotype, some tumor cells can boost the immunosuppressive effects of TAMs. For example, triple-negative breast cancer (TNBC) cells can promote M1-to-M2 conversion by secreting significant levels of granulocyte-colony stimulating factor [26]. M2-like TAMs increase hypoxia and aerobic glycolysis (the Warburg effect) in the TME, thus inhibiting the activation of CD4 + and CD8 + T effector cells, which are highly demanding glycolytically and metabolically [27]. This drives the expansion of Tregs, which prefer lipid oxidation over glycolysis [28]. TAMs, in contrast, express PD-L1 to deplete effector cells and induce PD-L1 expression in lung cancer cells by secreting IFN-γ via the Janus kinase/signaling transducer and activator of transcription 3 (JAK/STAT3) signaling pathway and the phosphoinositide 3-kinase (PI3K)/protein kinase B (Akt) signaling pathway [29]. In this way, TAMs achieve PD-1/PD-L1-dependent immunosuppression through a dual pathway. Recently, inhibitors of colony-stimulating factor-1 and its receptor (CSF-1R) have emerged as a viable solution to TAM-induced drug resistance. Targeted inhibition of CSF-1R signaling induces TAM redirection to the M1 phenotype and increases CD8 + T-cell infiltration [30]. Moreover, it has been demonstrated that combining anti-CSF-1R and anti-PD-1 monoclonal antibodies (mAbs) aids in total tumor eradication and is associated with a considerable increase in survival [31].

#### MDSCs

Unlike neutrophils and monocytes, MDSCs are the product of pathological activation of myeloid cells, such as that caused by persistent infections and cancers. These cells cause immunosuppression and support the progression and metastasis of tumors [32]. MDSCs produce reactive oxygen species, arginase 1, inducible nitric oxide synthase, and inhibitory factors like TGF-β during their expansion and activation, leading to T-cell apoptosis or T-cell receptor (TCR) nitrosylation as well as impaired NK cell function [33]. These actions directly affect the efficacy of PD-1 inhibitor-mediated immunotherapy. However, as our understanding of the MDSC role grows, it is clearer that they could provide novel targets for immunotherapies. According to one study, anti-CSF-1R and anti-ly6g antibodies specific for granulocytic MDSCs increased the therapeutic efficacy of anti-PD-1 by simultaneously targeting MDSCs and TAMs. This combination is more effective than ICI monotherapy for treating cholangiocarcinoma, a malignancy with a complex TME [34].

#### Mast cells (MCs)

MCs have a substantial role in TME regulation; this is beyond their involvement in allergic reactions. Histamine and heparin released by MCs impede tumor cell growth in certain malignancies, and MCs also enhance tumor invasion and angiogenesis by secreting chymotrypsin, trypsin, matrix metalloproteinase 9 (MMP-9), and VEGF [35]. It has also been demonstrated that MCs attract MDSCs and enhance the immunosuppressive properties of MDSCs [36]. Recent studies have shown that MCs are associated with anti-PD-1 resistance, and the combination of sunitinib or imatinib (TKIs used to deplete MCs) with anti-PD-1 is significantly more effective than monotherapy [37].

#### Cancer-associated fibroblasts (CAFs)

CAFs are one of the most abundant TME stromal components and are extensively involved in tumorigenesis and metastasis. In addition to promoting tumor metastasis and angiogenesis by secreting VEGFA, CAFs also secrete TGF-β, IL-6, and CC-chemokine ligand 2 to recruit immunosuppressive cells and suppress antitumor immunity [38]. Furthermore, CAFs interact with other immunosuppressive cells to combat antitumor immunity, making them a crucial component of the immunosuppressive cell network. In breast cancer, CAFs attract monocytes and induce them to differentiate into immunosuppressive PD-1 + M2-like TAMs [39]. Given the detrimental effects of CAFs on anti-cancer immunity, a growing number of studies are investigating CAFs as therapeutic targets. These cells promote malignant tumor progression by secreting WNT2, and targeted inhibition of WNT2 restores DC differentiation and enhances the efficacy of ICIs [40].

#### Tregs

In a physiological setting, regulatory T cells prevent the onset of autoimmune disorders by maintaining autoimmune tolerance. However, in a pathological setting, their immunosuppressive actions may encourage tumor cell immune evasion and undermine antitumor immunity. Treg cells can exert immunosuppressive functions through multiple pathways: Firstly, Treg cells can consume IL-2 through the high-affinity IL-2 receptor and secrete inhibitory cytokines such as IL-10, IL-35, TGF-β [41]; Secondly, CTLA-4 expressed on Treg cells can inhibit T cell activation by competitively inhibiting co-stimulatory signaling [42]; Thirdly, adenosine produced by CD39 and CD73 expressed on Treg cells interacts with the adenosine 2 A receptors (A2ARs) on the surface of T cells to prevent T cell growth [43]. In addition, Treg cells can co-construct immunosuppressive cellular networks by interacting with other immunosuppressive cells. For example, CAFs secrete TGF-β, which stimulates Treg cells. Treg cells can then produce TGF-β, triggering resident fibroblasts to differentiate into CAFs [44]. As ICB therapies are ineffective in most patients, immunotherapies targeting Treg cell depletion to address resistance has emerged. PD-1-deficient Treg cells were able to recover the autoimmune phenotype in mice and exhibited higher immunosuppressive activity than PD-1-expressing Treg cells [45]. This study confirms that loss of PD-1 expression or blockade of its signaling pathway increases the immunosuppressive potential of Treg cells, and explains why anti-PD-1 monoclonal antibody therapy leads to PD-1 + Treg cell-mediated resistance. Less than 10% of gastric cancer patients treated with anti-PD-1 mAbs have been reported to have rapid cancer progression, called highly progressive disease (HPD). HPD development is associated with enhanced suppressive activity of tumor-infiltrating PD-1 + Treg cells due to PD-1 blockade [46]. Therefore, targeting both PD-1 and CTLA-4 to deplete Treg cells may be able to prevent HPD.

#### Tumor-derived exosomes (TEXs)

Almost all mammalian cells can take up and secrete exosomes [47], which are extracellular vesicles that play an important role in cellular information exchange. Exosomes carry microRNAs (miRNAs), RNAs, proteins, and other substances that affect recipient cell signaling pathways or biological phenotypes, making their role in tumor progression a hot topic for research [48]. Recently, an increasing number of studies have shown that TEXs can initiate tumor immune evasion via the PD-1/PD-L1 axis, contributing to PD-1-mediated immunotherapy resistance. Yang and colleagues found that precursor and secretory exosomes of human breast cancer cells contain PD-L1, which can be transferred to other cells [49]. Immunoelectron microscopy and enzyme-linked immunosorbent assays showed that exosomal PD-L1 has the same membrane topology as cell surface PD-L1, and its expression level rises with IFN-γ secretion [50].

Exosomal PD-L1 is directly involved in immunosuppression and induces drug resistance. In vitro, small-cell lung cancer extracellular vesicles with PD-L1 can bind to TCRs and inhibit CD8 + T-cell activation. However, this effect can be reversed by anti-PD-L1 antibodies [51]. Also, TEXs indirectly resist immunotherapies by inducing PD-1/PD-L1 expression in other cells. For example, both HCC-derived and glioblastoma stem cell-derived exosomes induce PD-L1 expression on macrophages, promoting the secretion of immunosuppressive cytokines such as IL-6 and IL-10 [52, 53]. Of note, in addition to conferring immunotherapy resistance to tumor cells, some exosomes enhance the efficacy of ICIs by reprogramming the TME. It has been reported that exosome-like nanovesicles from M1 macrophages enhanced the antitumor effect of anti-PD-L1 antibody by repolarizing M2-like TAMs [54].

The role of exosomes as a bridge for intercellular communication in immunotherapy resistance is related to their cargo [55]. Under endoplasmic reticulum stress, exosomes secreted by HCC cells contain high levels of miR-23a-3p, which can upregulate PD-L1 expression by inhibiting PTEN in macrophages. This may be one of the reasons for the low response rates reported for some ICIs [56]. Human pancreatic cancer cells secrete exosomal LINC00460 (LncRNA), which promotes pancreatic cancer invasion and metastasis by inducing an increase in M2-like TAMs. However, silencing LINC00460 is associated with improved efficacy of anti-PD therapy [57]. Promotion of Src homology region 2-containing protein tyrosine phosphatase 2 expression through inhibition of miR-934 (exosomal circular ubiquitin-specific protease-7 derived from NSCLC cells) impaired CD8 + T-cell function and contributed to anti-PD-1 therapy resistance [58].

Because extracellular vehicles are a large family, the role of microparticles in immunotherapy should also be considered. Wei et al. demonstrated that mannose-modified macrophage-derived microparticles transporting metformin repolarized M2-like TAMs to M1-like TAMs, improving CD8 + T-cell infiltration, and leading to partial resolution of resistance to anti-PD-1 drug treatment [59].

In recent years, more researchers have realized that exosomes have the potential to be biomarkers for immunotherapy. While high levels of circulating exosomal PD-L1 before treatment are associated with T-cell depletion and poorer clinical outcomes, clinical responders showed an increase in circulating exosomal PD-L1 levels after treatment with the anti-PD-1 antibody pembrolizumab. This is likely because CD8 + T cells secrete more IFN-γ as they recover, thus increasing PD-L1, which is a marker of immune activation [50]. Another study found that plasma exosomal PD-L1 levels were significantly reduced in patients with complete or partial remission, but increased in patients with progressive melanoma or NSCLC after two months of treatment with either the anti-PD-1 antibody nivolumab or pembrolizumab [60]. This paradox may be due to the patient’s plasma exosomes being a heterogeneous mix of tumor- and non-malignant cell-derived exosomes [61]. Nevertheless, it remains useful to detect PD-L1 levels in exosomes to help select treatment options and maximize patient benefits. Also, targeting TEXs is a new strategy for immunotherapies. Macitentan inhibits extracellular vesicle PD-L1 secretion by targeting endothelin A-type receptors, thus activating T cells. Combined with anti-PD-L1 antibodies, this treatment could enhance antitumor immune responses [62].

### Gut microbiota

The gut microbiota and its metabolites, the body’s largest and most complex ecosystem, greatly impact immunity. As part of the intestinal immunological barrier, the gut microbiota maintains metabolism and interacts with the host’s innate immunity [63]. Deshmukh et al. demonstrated that antibiotic exposure reduces the number of circulating neutrophils in the bone marrow of pregnant mice’s offspring, which is associated with a decrease in the neonatal gut microbiota [64]. Similarly, innate immunity regulates microbiome composition. Innate lymphoid cells generate IL-22, which regulates *Alcaligenes* spp. spread and inflammation [65]. More than that, the role of gut microbiota for tumor immunity should not be overlooked. Research has demonstrated the diverse effects that different bacterial genera play in various types of tumors. *Fusobacterium nucleatum* accelerates CRC [66]. In contrast, *Clostridium butyricum* and *Bacillus subtilis* may cause CRC cell apoptosis [67].

**Fig. 2:**
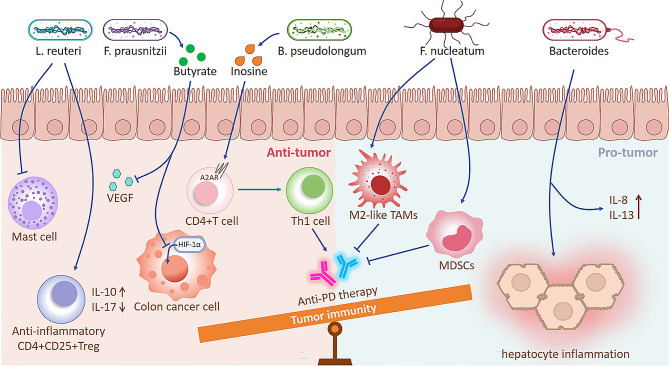
Role of the gut microbiota in tumor immunity. The gut microbiota and its metabolites are capable of exerting anti/pro-tumour effects through multiple pathways. *L. reuteri* Lactobacillus reuteri, *F. prausnitzii* Faecalibacterium prausnitzii, *F. nucleatum* Fusobacterium nucleatum, *HIF-1α* Hypoxia-inducible factor-1α, *B. pseudolongum* Bifidobacterium pseudolongum

Indeed, gut microbiota modulate tumor immunity mostly via the TME. This explains why certain “beneficial genera” are potential immunotherapy targets. On the one hand, the gut microbiota is able to regulate cells in the TME. For example, *Fusobacterium nucleatumn* was able to enrich MDSCs and M2-like TAMs in mice TME compared to controls, resulting in a pro-cancer impact [66]. On the other hand, gut microbial metabolites also modulate TME. Intestinal *Bifidobacterium pseudolongum* produces inosine, which stimulates T helper type 1(Th1) cells by binding to T cell A2ARs, boosting anti-PD-1 or anti-CTLA-4 effectiveness [68] (Fig. 2).

Several researches have shown how particular bacterial species work in concert with ICB, providing fresh perspectives on how to maximize ICB’s effectiveness. Sivan et al. demonstrated that for melanoma mice, oral administration of *Bifidobacterium* significantly magnifies the efficacy of PD-L1 blockade [69]. Moreover, simultaneous oral gavages with *Akkermansia muciniphila* and *Enterococcus hirae* 13,144 enhanced the growth inhibition of lung cancer by anti-PD-1 antibodies [6]. On the contrary, disturbances in gut ecology impair the efficacy of ICB. Oral antibiotics, in addition to anti-PD-1/PD-L1 antibodies, reduced patients’ progression-free survival (PFS) and/or OS [6](Fig. 2).

Although more researches are needed to elucidate the relationship between certain bacterial species and tumor immune therapy, changing the abundance of gut microbiota to regulate TME and constructing bioengineered bacteria to enhance the efficacy of immunotherapy is a new direction for cancer treatment.

### Epigenetics and immune escape

Epigenetic regulation is associated with tumor development and drug resistance. DNA methylation is the process by which DNA methyltransferases (DNMTs) obtain methyl groups at the fifth carbon atom of DNA cytosines, resulting in gene silencing. Histone acetylation refers to the structural opening of certain genes, allowing access for transcription and boosting expression; it is dually regulated by histone acetyltransferases and histone deacetylases (HDACs). Tumor cells can trigger cancer growth via epigenetic alterations. Regional hypomethylation in tumor cells can lead to decreased genomic stability, increased mutation rate, and oncogene transcription. Furthermore, high methylation or deacetylation can inactivate tumor suppressor genes [70].

Epigenetic regulation may cause immune escape. Luo et al. demonstrated that the major histocompatibility complex (MHC) class I gene is methylated in breast cancer and that this suppression can be reversed by the hypomethylating drug guadecitabine [71]. Epigenetics has also been shown to reprogram the TME, especially for the activation of immunosuppressive cells. HDAC9 converts macrophages to a pro-inflammatory phenotype (M1-like TAMs) [72]. Moreover, the establishment of Treg cell-type CpG hypermethylation is critical for Treg spectral stability and complete suppressive function [73].

A few epigenetic drugs such as the DNMT inhibitor azacitidine and the HDAC inhibitor vorinostat have recently been approved for myelodysplastic syndromes and cutaneous T-cell lymphoma. Growing evidence shows that some epigenetic drugs modulate the TME and enhance antitumor effects. For example, the HDAC inhibitor entinostat can reduce the number of MDSCs. In 4T1 tumor-bearing mice, a combination of entinostat, anti-PD-1, and anti-CTLA-4 antibodies strengthened the antitumor effect and eradicated the tumor [16]. Furthermore, by knocking down Brd4, JQ1 (a bromodomain and extra-terminal domain inhibitor) reduced PD-L1 expression on the surface of tumor cells. When combined with anti-PD-1 antibodies, it significantly improved survival rates in mice with lymphoma compared with monotherapy [74]. In a randomized phase 2 trial in patients with advanced chemorefractory metastatic microsatellite stable/proficient mismatch repair colorectal cancer, triple combination therapy with anti-PD-1 mAbs plus HDAC inhibitor and anti-VEGF mAbs significantly enhanced CD8 + T-cell infiltration in the TME, improving the overall response rate to 44.0% [75]. A combination of pembrolizumab and vorinostat for primary mediastinal B-cell lymphoma demonstrated complete and overall response rates of up to 80%, which was significantly superior to monotherapy [76]. Given its intimate association with both tumor formation and immune evasion, epigenetics may be the target of future immunotherapies. The development of specifically designed epigenetic drugs will increase the opportunities for using combination treatments.

### Activation of immune checkpoints

**Fig. 3:**
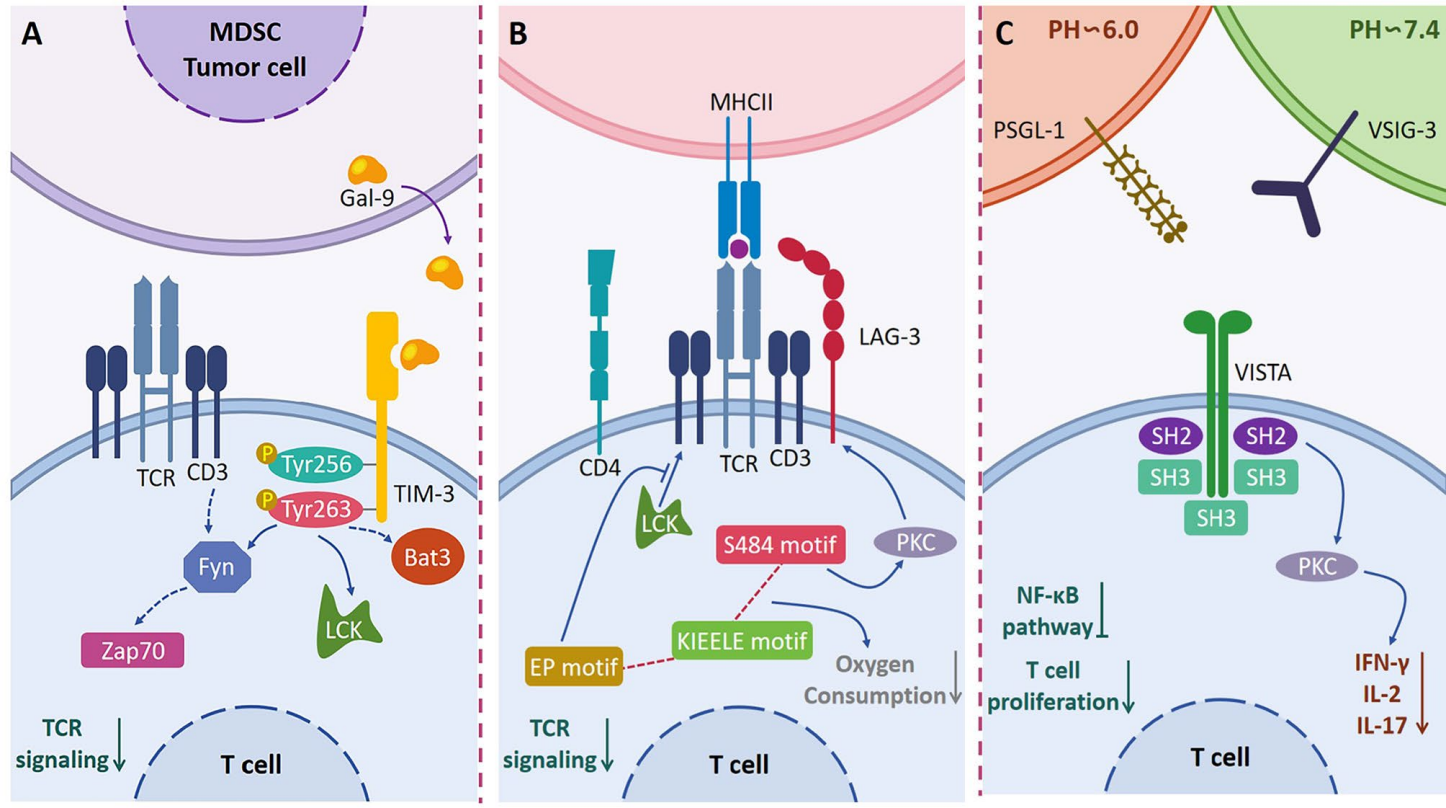
Mode of action of TIM-3, LAG-3, VISTA signaling pathways. (**A**) TIM-3 binds to Gal-9 and induces Tyr256 and Tyr263 phosphorylation, releasing Bat3, which regulates Fyn and LCK tyrosine kinases, thereby inhibiting TCR signalling. (**B**) The S484 motif upregulates the surface expression of LAG-3 through PKC signalling; KIEELE is responsible for regulating downstream inhibitory signalling; EP motifs interfere with T cell activation by blocking CD3/Lck interactions. (**C**) VISTA binds to PSGL-1 in acidic environments and to VSIG-3 in physiological environments, inhibiting T cell function and proliferation

Since the approval of ipilimumab for treating metastatic melanoma, immunotherapies focused on targeting co-inhibited receptors have ushered in a new era of cancer treatment [1]. In addition to CTLA-4 and PD-1, numerous new co-inhibitory receptors have emerged in the past two decades, explaining the resistance of some ICIs. At the same time, a deeper understanding of the mechanism of ICIs will provide new candidate targets for immunotherapy (Fig. 3).

#### TIM-3

TIM-3, which is expressed on the surface of Th1 cells, Th17 cells, macrophages, and DCs, is defined as a co-inhibitory receptor. As a negative regulator of the immune action of Th1 cells, TIM-3 is important for controlling the progression of autoimmune diseases [77], and as an immune checkpoint, it has received much attention for its role in antitumor immunity. TIM-3 drives immunosuppression by binding to ligands. Similar to the binding of PD-L1 to PD-1, galectin (Gal)-9, a high-affinity TIM-3 ligand secreted by tumor cells, binds to TIM-3 and initiates a negative feedback loop that induces T-cell apoptosis [8]. Yasinska et al. discovered the fibronectin leucine-rich transmembrane protein 3-neuronal receptor latrophilin-TIM-3-Gal-9 pathway in several primary malignancies, including breast cancer. Blocking anticancer effector cell activation keeps tumor cells alive [78], and TIM-3 expression amplifies immunosuppressive cell activities. It has been shown that TIM3 + Tregs express more IL-10, perforin, and granzymes A and G than TIM3- Tregs [79]. This suggests that TIM-3 inhibition suppresses regulatory T-cell activity and helps exhausted T cells recover.

TIM-3 expression is closely related to anti-PD therapy resistance and thus provides an optional target for dual immunotherapies. Koyama et al. demonstrated that TIM-3 expression is upregulated in anti-PD-1-resistant tumors. Combination therapy with anti-PD-1 antibodies makes sense because anti-TIM-3 antibodies improve T-cell function and reduce levels of cytokines that promote tumor growth after the failure of anti-PD-1 antibodies [80]. Targeted inhibition of TIM-3 ligand Gal-9 is another promising therapeutic strategy. As Yang et al. demonstrated, in EMT-6 mouse TNBC, combination therapy with anti-Gal-9 and anti-PD-L1 antibodies achieved better survival rates than either monotherapy [81]. Anti-TIM-3 antibodies are also superior candidates for controlling immune-related adverse events. The activation of T cells, especially auto-reactive T cells, may lead to immune-related adverse events [82]. However, TIM-3 is selectively expressed on T cells that produce IFN-γ [83]. Therefore, targeting TIM-3 has a lower probability of causing adverse reactions compared with targeting PD-1.

TIM-3-induced immunosuppression is present in a variety of immune cells, including CD8 + T cells and Tregs. Consequently, targeted blockade of TIM-3 is important for reprogramming the TME. Given that PD-1 blockade may lead to TIM-3 upregulation, combination therapies targeting TIM-3 should greatly improve the efficiency of immunotherapy.

#### LAG-3

LAG-3 is expressed on activated human T cells and NK cells and is structurally similar to CD4 [84]. LAG-3 competes with CD4 to bind to MHC class II molecules, which affects the function of CD4 + T cells. Hemon et al. demonstrated that LAG-3 molecules protect MHC class II-positive melanoma cells from Fas-induced apoptosis by activating mitogen-activated protein kinase (MAPK)/Erk and PI3K/Akt pathways [85]. The inhibitory effect of LAG-3 on CD8 + T cells is then dependent on binding to Gal-3. Wildtype CD8 + T cells showed a significant decrease in IFN-γ production in the same exogenous Gal-3 environment, while LAG-3 knock-out cells were unaffected [9]. LAG-3 interacts with the lectin LSECtin on B16 melanoma cells to inhibit effector T cell IFN-γ secretion [86].

The above studies suggest that LAG-3 inhibits antitumor immunity through multiple pathways. In the murine TME, LAG-3 is often co-expressed with PD-1. For example, PD-1 + LAG-3 + CD8 + T cells are the mainstay of CT26 tumor-infiltrating lymphocytes [87]. Hence, compared with monotherapy, combination therapies are noticeably more successful. In comparison with monotherapy, combined inhibition of LAG-3 and PD-1 increased full tumor regression in approximately 50% of Sa1N fibrosarcoma mice [88].

It is not entirely clear how LAG-3 induces T-cell exhaustion, but LAG-3 and PD-1 are functionally synergistic. Combined blockade of LAG-3 and PD-1 can reverse the lack of response to monotherapy and has potential for the treatment of refractory tumors.

#### VISTA

Mainly expressed on hematopoietic cells, VISTA is highly homologous to PD-L1 [89]. In recent years, the role of VISTA as an immune checkpoint with therapeutic potential in the TME has been a hot topic. Wang et al. demonstrated that V-Set and Immunoglobulin domain containing 3 (VSIG-3) is one of the ligands of VISTA and the interaction between the two inhibits the secretion of cytokines such as IFN-γ, IL-2, and IL-17 by T cells [10]. In acidic ph environments, VISTA acts as an inhibitory receptor by binding P-selectin glycoprotein ligand-1 to inhibit IFN-γ production and nuclear factor kappa B(NF-κB) phosphorylation [90]. Up-regulation of VISTA expression enhances the inhibitory activity of MDSCs under hypoxic conditions [91].

As the role of VISTA as an inhibitory immune checkpoint becomes clear, VISTA gradually involved in combination therapies. Johnston et al. demonstrated that the anti-tumor effect achieved by co-blocking VISTA and PD-1 is significantly better than monotherapy. Combined therapies increased T cell infiltration and decreased the expression of PD-1, LAG-3 and TIM-3 on T cell surface [90]. Of note, VISTA has been proved to be a potential contributor to resistance to anti-PD-1 and anti-CTLA-4 therapies. After ipilimumab treatment, VISTA expression was significantly up-regulated, mainly on the surface of CD4 + and CD8 + T cells and CD6 + macrophages [92]. This provides a new way to solve the drug resistance of immunotherapies targeted by PD-1 and CTLA-4.

Interestingly, despite numerous studies supporting the idea that VISTA is an inhibitory immunity checkpoint, the definition of VISTA remains controversial. In a cohort analysis of patients with esophageal adenocarcinoma, those with VISTA + tumor-infiltrating leukocytes(TILs) had a greater overall survival in the early tumor stage than those without VISTA + TILs [93]. Nevertheless, VISTA remains a potential target to address the inefficacy of anti-PD-1/PD-L1 antibodies and further research is needed to demonstrate the efficacy and safety of inhibiting VISTA.

## Combined antitumor immunotherapy strategies for tumor resistance

**Fig. 4:**
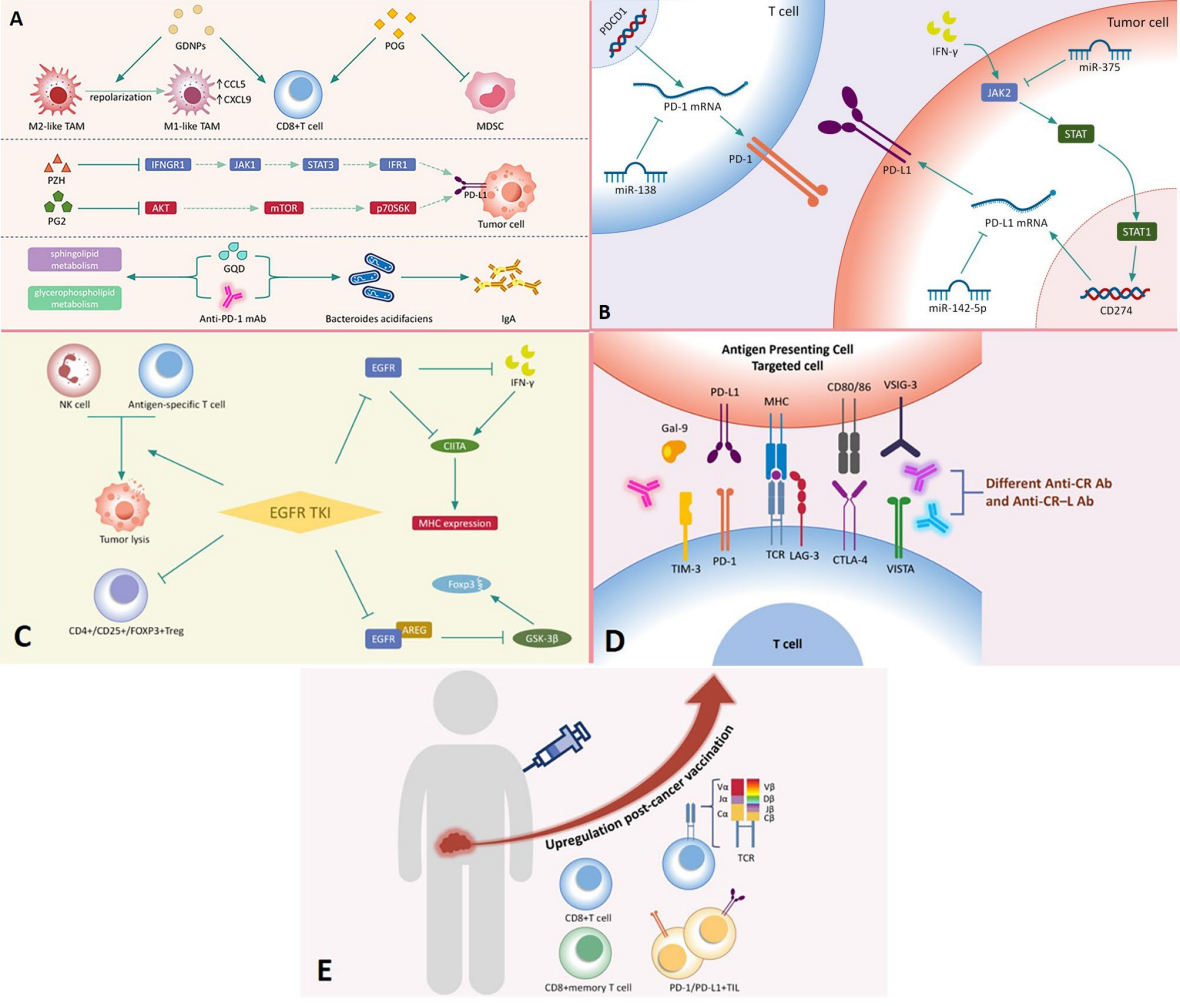
An overview of underlying mechanisms of combination therapy. (**A**) Various TCM ingredients can reprogram TME, regulate PD-L1 expression, modulate metabolism and enrich “beneficial bacteria” that can promote IgA production. (**B**) Non-coding RNAs can directly or indirectly regulate PD-1/PD-L1 expression. (**C**) Targeted therapies (such as EGFR TKI) promote tumor cell lysis, MHC expression and Foxp3 degradation. (**D**) Combined blockade of checkpoint receptors (CR) and their ligands (CR–L). (**E**) Personalized cancer vaccines are able to upregulate a wide range of immune cells and TCR-β clonotypes

ICIs have been the most successful therapeutic strategy in recent years. While anti-PD therapies are a crucial component of cancer immunotherapy, only a small percentage of patients respond to these drugs [94]. There is a plethora of resistance mechanisms, such as upregulation of other immune checkpoints, disturbances in gut microbiota, epigenetic alterations, and an immunosuppressive TME (Fig. 4). These factors have a considerable impact on the therapeutic efficacy of anti-PD therapies. Because multiple antitumor immunity pathways are activated, combination therapy strategies are becoming increasingly popular in combating drug resistance. Since combining anti-PD treatments with additional therapies can enhance the efficacy of immunotherapy, the FDA has approved some of these combinations, for example, with CTLA-4 inhibitors or angiogenesis inhibitors [14, 95].

### PD-1 mAb combined with traditional Chinese medicine (TCM)

Research has led to a developing interpretation of TCM’s mechanism of action from a clinical medicine perspective. Given that a subset of TCMs contribute to anti-tumor immunity, many studies have focused on combining TCMs with anti-PD-1 antibodies (Fig. 4A**)**. Some TCMs target the TME to exert anti-cancer effects. Ginseng derived nanoparticles (GDNPs) can repolarize TAMs, promote CCL5 and CXCL9 secretion, and increase tumor-infiltrating CD8 + T cells. GDNPs combined with anti-PD-1 mAb inhibit tumor progression better than monotherapy [96]. Of note, some TCMs also exert anti-tumor effects by regulating the PD-1/PD-L1 axis. Pien Tze Huang (PZH) inhibits IFNGR1-JAK1-STAT3-IRF1 signaling pathway to inhibit IFN-γ-induced up-regulation of PD-L1 expression. Combined with anti-PD therapies, drug resistance generated by up-regulation of PD-L1 expression in tumor cells can be solved [97].

However, the components of TCMs are complex and further researches are needed to confirm which specific compounds support the fight against cancer. The control of dosage and toxicity is also the key to expand the application prospect of TCMs.

### Anti-PD-1 mAbs combined with non-coding RNAs

With the development of whole genome sequencing technology, more and more studies have shown that non-coding RNAs can regulate the PD-1/PD-L1 axis. Among these, miRNAs have been most extensively investigated. Some miRNAs exert regulatory effects by directly targeting the 3’ untranslated region (3’UTR) of PD-1/PD-L1 (Fig. 4B**)**. For example, miR-15a-5p and miR-16-5p reduce PDCD1 (the gene encoding PD-1) expression at the protein level by binding to target sites within the 3’UTR of the gene [98]. Some miRNAs also regulate PD-1/PD-L1 expression indirectly by regulating other signaling pathways. MiR-375 downregulated JAK2 expression at the post-transcriptional level; this enzyme is involved in the IFN-γ-induced up-regulation of PD-L1 expression [99].

Similarly, long non-coding RNAs (lncRNAs) have regulatory effects on the PD-1/PD-L1 axis. LncRNA HOXA transcript at the distal tip increases IL-6 secretion by increasing binding of the transcription factor c-Jun to the IL-6 promoter, leading to increased IL-6-mediated PD-L1 expression in neutrophils [100]. There are also many studies on the regulation of PD-1/PD-L1 expression by circular RNAs. For example, circIGF2BP3 upregulates plakophilin 3 (PKP3) expression by mopping up miR-328-3p and miR-3173-5p. In addition, PKP3 is involved in stabilizing OTUB1 mRNA and upregulating its expression. As a deubiquitinase that stabilizes PD-L1, OTUB1 levels positively correlate with PD-L1 protein levels. Finally, circIGF2BP3 overexpression inhibits antitumor immunity by upregulating PD-L1 expression [101].

Moreover, non-coding RNAs broadly affect immunotherapy. MiR-21-3p-loaded gold nanoparticles induced lipid peroxidation production and ferroptosis in melanoma cells by directly targeting thioredoxin reductase 1, significantly enhancing the therapeutic effect of anti-PD-1 antibodies [102].

To date, the following approaches have been used to target miRNAs. Firstly, synthetic miRNA mimics compensate for the absence of miRNAs with tumor-suppressor functions by synthesizing small double-stranded RNA molecules that match the sequence of the corresponding miRNAs, enabling them to inhibit downstream mRNAs. Secondly, antimiRs, also known as inhibitors of miRNAs, block the inhibition of mRNAs by binding to the target miRNA [103]. Thirdly, small molecule inhibitors of miRNAs modulate miRNA function by targeting miRNA biosynthesis processes, such as binding to the miRNA biosynthesis protein TAR RNA-binding protein 2. Lastly, because extracellular vesicles play an important role in miRNA transport, interfering with exosome secretion and delivery can also affect miRNA function [104].

Finding effective targets is crucial for balancing toxicity and efficacy because non-coding RNAs can control the expression of the PD-1/PD-L1 axis and other upstream and downstream targets. The selection of vectors with low toxicity, high stability, and the ability to provide targeted transport is critical for the clinical application of non-coding RNAs. Drug delivery systems for miRNA-based therapeutics are constantly being updated. In addition to the commonly used lipid nanoparticles, there are various drug-delivery systems such as adenoviral vectors, poly(lactide-co-glycolide) particles, and EnGeneIC Delivery Vehicle [105–108]. Indeed, the difficulty of targeted delivery and the immune response of the individual make it challenging to translate these promising vectors to the clinic. However, recent studies have shown that plant-derived exosome-like nanoparticles are rich in surface sites that are easily accessible through specific binding to enable targeting of tumor sites [109]. The flexible use of non-coding RNAs as therapeutic targets and interventions should help enhance the efficacy of ICIs.

### Anti-PD-1 mAbs combined with targeted therapy

Despite remarkable advances in oncology treatment, many patients continue to respond poorly to targeted therapies. For example, sorafenib treatment in individuals with advanced HCC only extends survival by approximately 3 months [110]. Therefore, it is urgent to address the issue of resistance to targeted therapies.

As a multiple TKI, sorafenib inhibits the serine/threonine kinases c-Raf (Raf-1) and B-Raf, VEGFR, platelet-derived growth factor receptor, and cytokine receptor c-KIT [111]. According to Liang et al., continuous sorafenib treatment raises levels of the hypoxia-inducible factor 1 (HIF-1α) protein, which causes hypoxia-mediated resistance to sorafenib [112]. The immunosuppressive microenvironment additionally triggers sorafenib resistance. Sorafenib-induced hypoxia causes immunosuppression by increasing the expression of M2-like TAMs, Tregs, and PD-L1 in the TME [113]. Furthermore, activation of PD-L1/STAT3/DNMT1 also contributes to the development of sorafenib resistance in HCC [114]. Anti-PD-1 mAbs can effectively inhibit sorafenib-induced PD-1/PD-L1 upregulation and tumor angiogenesis. Shigeta et al. discovered that inhibiting PD-1 promotes CD4 + cell-mediated blood vessel normalization and reduces hypoxia [115]. Therefore, anti-PD-1 mAbs not only alleviate the immunosuppression caused by sorafenib-induced PD-1/PD-L1 overexpression, but also help restore normal blood vessels. This is expected to become a new method of addressing sorafenib resistance.

Similarly, the long-term efficacy of EGFR TKIs depends on whether acquired drug resistance develops. T790M mutation, a threonine-to-methionine transition at amino acid position 790 in exon 20 of EGFR, is the most common acquired resistance mechanism. The resulting spatial blockage affects the binding of EGFR TKI and reduces its therapeutic efficacy [116]. EGFR-T790M mutations also increase PD-L1 expression via PI3K/Akt, MAPK, and NF-κB signaling pathways, promoting tumor immune evasion [117]. MET amplification activates the PI3K/Akt pathway by maintaining ERBB3 phosphorylation, resulting in resistance to the first-generation EGFR TKI gefitinib [118]. Interestingly, activation of the hepatocyte growth factor/c-MET axis was found to mediate PD-L1 upregulation via PI3K/Akt and MAPK signaling pathways. High PD-L1 expression was associated with therapeutic benefit from anti-PD-1/PD-L1 treatment in patients with lung cancer and EGFR-TKI resistance [117]. Therefore, EGFR-TKI resistance caused by these pathways can be partially resolved by blocking the PD-1/PD-L1 axis.

As research has progressed, resistance mechanisms have been identified for anti-angiogenic drugs. Schmittnaegel et al. demonstrated that dual angiopoietin-2 and VEGF inhibition activates IFN-γ-expressing CD8 + CTLs while impairing tumor angiogenesis. However, this causes a rise in IFN-γ-mediated PD-L1 expression, inducing immunosuppression and weakening the original antitumor effect. Increasing PD-1 blockade reverses this and comprehensively activates antitumor immunity [119]. Nevertheless, Zheng et al. demonstrated that by encouraging CD8 + T lymphocyte growth, anti-PD-1 treatment may reduce tissue hypoxia and improve vascular perfusion [120]. VEGF promotes PD-1 expression on the T-cell surface by activating the VEGFR2-phospholipase C γ/calcineurin/nuclear factor of the activated T-cell signaling pathway. Targeting PD-1 and VEGF together should provide a greater therapeutic impact in tumors with high VEGF levels [121]. This suggests that anti-PD-1 antibodies are promising candidates for patients with resistance to anti-angiogenic agents.

Targeted therapies also enhance the antitumor effects of anti-PD-1 antibodies in several ways. Hage et al. demonstrated that sorafenib exerts antitumor effects by inducing macrophage pyroptosis and activating cytotoxic NK cells. Sorafenib also modulates the TME towards a pro-inflammatory, antitumor state [122], providing evidence for sorafenib use in immunotherapy combinations.

Similarly, EGFR TKIs also modulate both the TME and the functional state of immune cells in concert with anti-PD therapies. EGFR TKIs reduced the ratio of CD4+/CD25+/FOXP3 + regulatory T cells in the TME [18], making the immune TME favorable for anti-PD treatments. Furthermore, EGFR TKIs enhanced the induction of MHC class I and MHC class II molecules by IFN-γ, inhibiting immune evasion by tumor cells [19]. The above study emphasizes the beneficial effects of EGFR TKIs in combating tumor immunity and suggests that these drugs could enhance the clinical benefits of anti-PD therapy (Fig. 4C**)**.

Anti-angiogenic agents are also considered to be allies of anti-PD-1 antibodies. Similarly, low-dose anti-VEGFR-2 antibodies may reprogram the TME by decreasing MDSC recruitment, encouraging TAM M1-to-M2 conversion, and boosting CD4 + and CD8 + T-cell infiltration [12]. Normalizing tumor vasculature reduces Treg-mediated immunosuppression and boosts DC antigen presentation [123]. Not only does the elimination of abnormal blood vessels help to restrict tumor growth, but also promotes the formation of an antitumor TME, indicating that anti-angiogenic agents can boost anti-PD treatments.

To date, numerous studies have confirmed that combining targeted drugs with anti-PD-1 antibodies induces stronger antitumor immunity. However, not all combination treatment strategies result in good clinical outcomes. Both the TATTON and CAURAL clinical trials failed because of the high incidence of interstitial pneumonitis caused by combining the third-generation EGFR-TKI osimertinib with the anti-PD-L1 antibody durvalumab. Similarly, interstitial pneumonitis is a known adverse event of combining osimertinib and nivolumab [124]. Improved management of adverse events is essential for combination therapy. In a phase 3 trial for patients with advanced HCC, 30-month OS was approximately 39% with a lenvatinib and pembrolizumab combination, compared with approximately 31% with lenvatinib alone. Notably, the proportion of patients experiencing grade 3–5 adverse events was higher for combination therapy than for monotherapy [125]. Therefore, combination therapy is only justified if the risks are controllable and the efficacy is significantly superior to that of monotherapy. Nevertheless, as a commonly used cancer treatment in clinical practice, targeted therapy has great promise in combination with anti-PD therapy. Exploration of the underlying mechanisms of combination therapies will help guide both patient section and medicine use.

### Dual immunotherapy

As more inhibitory receptors are being explored as possible targets for immunotherapies, the poor response rate to ICI monotherapy has raised the question of whether simultaneous blockade of two or more immune checkpoints might improve clinical outcomes (Fig. 4D**)**. Unlike PD-1, CTLA-4 competes with CD28 for B7 receptors on antigen-presenting cells to inhibit T-cell proliferation. Targeted CTLA-4 inhibition can restore T-cell proliferation and IL-2 production, enhancing antitumor immunity [126]. In addition to ipilimumab, which has been approved by the FDA for use in metastatic melanoma, many studies have confirmed that CTLA-4 inhibition is beneficial for glioblastoma multiforme [127], acute myeloid leukemia, and Hodgkin’s lymphoma [128].

Most patients receive little benefit from ICI monotherapy. Pembrolizumab, nivolumab, and ipilimumab have response rates of less than 50% in patients with metastatic melanoma when used as monotherapy [129]. However, dual blockade of CTLA-4 and PD-1 is considered a promising immunotherapeutic strategy. In patients with NSCLC, nivolumab plus ipilimumab demonstrated a longer median progression-free survival (PFS) and higher security compared with nivolumab monotherapy [130]. Notably, the combination of PD-1 inhibitors with CTLA-4 inhibitors also had superior antitumor effects when used to treat metastatic esophagogastric cancer [131] and metastatic sarcoma [132].

Because of the clear therapeutic advantages of inhibiting both PD-1 and CTLA-4, combining other ICIs is considered an effective therapeutic strategy. Combining the anti-TIM-3 antibody sabatolimab and the anti PD-1 antibody spartarizumab initially activated antitumor activity in advanced solid tumor patients who did not respond to monotherapy [133]. The FDA approved nivolumab plus the anti-LAG-3 antibody relatlimab as a fixed-dose immunotherapy for unresectable or metastatic melanoma in March 2022 [134]. Of note, some recent studies have reported further activation of antitumor immunity by simultaneously targeting the TME and PD-1. Bempegaldesleukin, an IL-2 pathway agonist, increases the proliferation and infiltration of CD8 + T cells, NK cells, and PD-1/PD-L1 expression in the TME. A combination of bempegaldesleukin and nivolumab significantly prolonged PFS in patients with metastatic melanoma [135]. Notably, the emergence of bispecific antibodies has provided new insights into the resolution of immunotherapy resistance. By 2023, nine bispecific antibodies had been approved by the FDA [136]. Consequently, research is increasingly focused on developing bispecific antibodies. For example, Yuwen et al. designed a bispecific antibody that blocks PD-1/PD-L1 binding while activating 4-1BB (a member of the tumor necrosis factor receptor superfamily); this leads to reprogramming of the TME and potent activation of antitumor immunity [137]. Furthermore, the novel bispecific monoclonal antibody ZGGS15, which competitively inhibits the binding of LAG-3 to MHC class II and the binding of T cell immunoreceptor with immunoglobulin and ITIM domain (TIGIT) to CD155, significantly suppressed tumor growth in melanoma-bearing mice with a weak response to anti-PD-1 treatment [138].

Considerable research suggests that dual blocking of immune checkpoints greatly enhances patient response rates and survival, signifying a breakthrough in immunotherapy. However, immune-related adverse effects are key to combination therapy effectiveness. Predicting and managing these events should be a new focus for research.

### Anti-PD-1 mAbs combined with personalized cancer vaccines

**Fig. 5:**
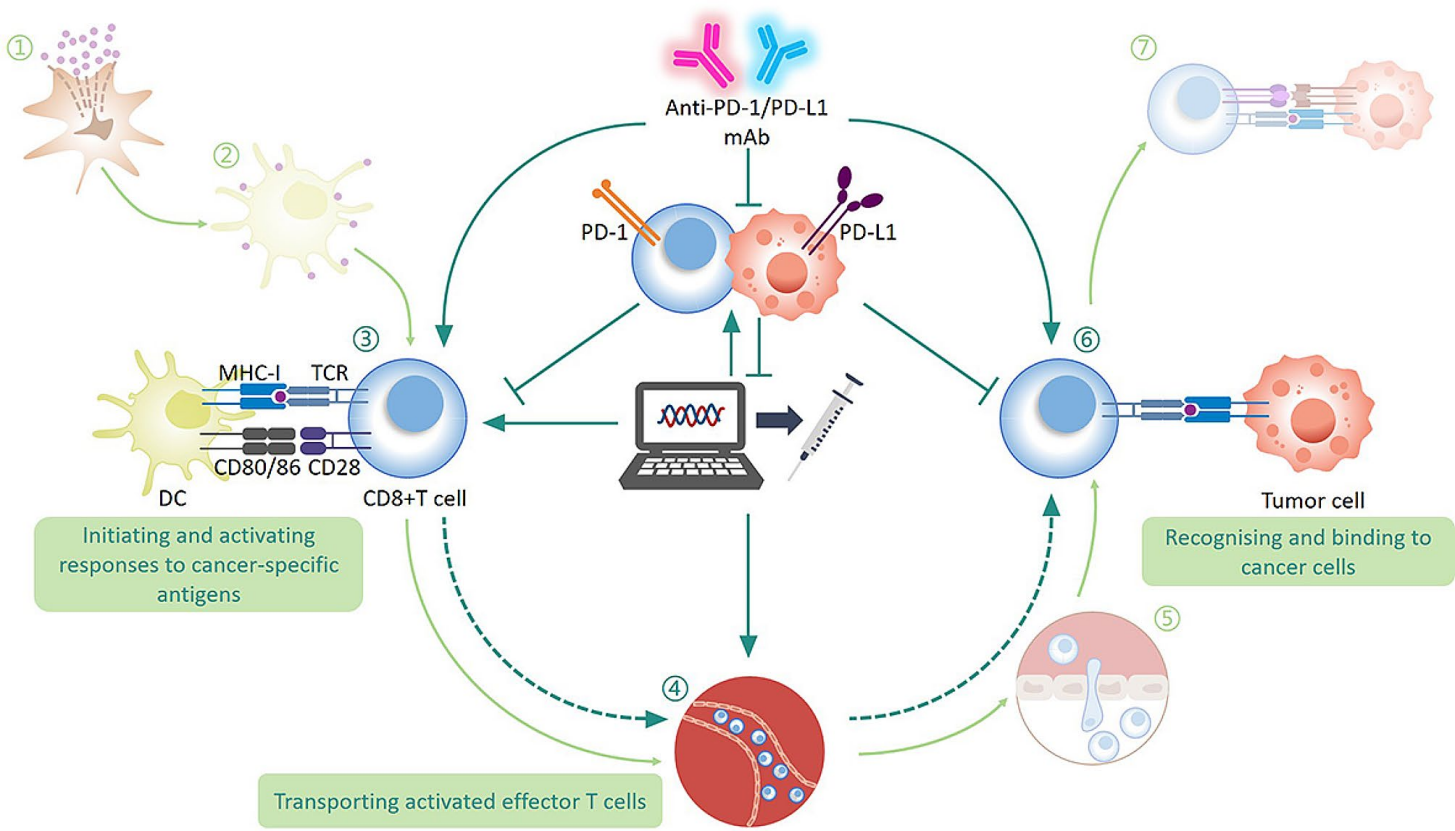
The role of personalized cancer vaccines and anti-PD therapy in the “Cancer-Immunity Cycle”. Personalized Cancer vaccines boost steps 3 and 4 but induce elevated PD-1/PD-L1 in TME. Elevated PD-1/PD-L1 in turn induces potent immunosuppression, impairs step 3 and 6, limiting the efficacy of personalized cancer vaccines. Anti-PD therapy can overcome immunosuppression in steps 3 and 6. Combined application of personalized cancer vaccines and anti-PD therapy can facilitate an efficient cancer “Cancer-Immunity Cycle”, promoting tumor regression

Advances in genome sequencing technologies and protein mass spectrometry have led to widespread use of personalized cancer vaccines. “Neoantigens” are produced by mutations in cancer cells and can be identified by autologous T cells without central tolerance, making them an ideal therapeutic target. T cells recognize “neoepitopes” as mutant epitopes generated by the neoantigen [139]. Personalized vaccines can induce and activate CD4 + T helper 1 cells and CD8 + T cells to target neoantigens and kill cancer cells, resulting in the release of cancer antigens and positively promoting the “cancer-immunity cycle” [140] (Fig. 4E**)**. Chen et al. divided the cancer-immunity cycle into seven steps: (1) neoantigen capture; (2) antigen presentation; (3) initiating and activating responses to cancer-specific antigens; (4) transporting activated effector T cells; (5) effector T cell infiltration; (6) recognizing and binding to cancer cells; and (7) killing cancer cells. Immunostimulatory and suppressive factors co-regulate this cycle and, respectively, their accumulation enhances and weakens anticancer immunity [141] (Fig. 5).

Theoretically, combining immunotherapies is supported by understanding the cancer-immunity cycle. In “cold” tumors that lack T-cell infiltration, the cycle has difficulty reaching the third step, resulting in little benefit from ICIs [96]. However, personalized cancer vaccines can directly activate and expand neoepitope-specific T cells, thus improving infiltration [139]. For example, neoantigen vaccination of patients with melanoma led to the recruitment of a broad T-cell repertoire and significantly increased CD8 + T-cell infiltration compared with that before vaccination [142]. Notably, neoepitope-specific T cells were PD-1+, and PD-L1 was upregulated in the TME following vaccination [142, 143]. This finding suggests that personalized vaccines provide a suitable TME for PD-1 inhibitors and can facilitate their induced antitumor immunity. Intriguingly, neoantigenic vaccines can upregulate PD-1/PD-L1 expression, leading to immunosuppression that affects steps three and six of the cancer-immunity cycle, and limiting vaccine-induced tumor regression [139, 143]. However, inhibiting PD-1/PD-L1 can address this problem. Following pembrolizumab treatment, Ott et al. demonstrated that two melanoma patients with lung metastases achieved complete tumor regression after neoantigen vaccination [144] (Fig. 5).

A growing number of studies have demonstrated that combining PD-1/PD-L1 inhibitors and neoantigen vaccines significantly improves antitumor immunity, making it a feasible strategy for combination therapy. In a murine model of aggressive glioblastoma, combination therapy with neoantigen vaccines and PD-L1 inhibitors significantly enhanced the antitumor effect compared with monotherapy, resulting in long-term survival for 60% of mice [145]. Furthermore, neoantigen vaccine plus PD-1 inhibitor treatment provided sustained tumor regression, enhanced CD8 + memory T cell production, and inhibited HCC metastasis and recurrence compared with monotherapy [146].

As data science advances, cancer vaccines are targeting “neoantigens” rather than tumor-associated antigens [147]. Of note, neoantigen vaccines are truly personalized therapies, with the added benefits of multitargeting and safety. Similar to immunotherapies, both neoantigen vaccines and anti-PD therapies can generate antitumor effects by remodeling the TME and enhancing the cancer-immunity cycle. Because of their different mechanisms of action, combination immunotherapies can stimulate more powerful antitumor immunity and become a widely applicable cancer treatment strategy.

## Biomarkers for immune therapy response and resistance

As discussed above, the effectiveness of immunotherapy against tumors is limited by drug resistance. Although the emergence of combination therapy is a promising solution to this problem, the implementation of combination therapy remains challenging. Can we determine whether patients are resistant to anti-PD therapy before taking medication? If we decide to use combination therapy, can we determine which combination therapy the patient is sensitive to before treatment? The answers to these questions depend on establishing a comprehensive system of biomarkers to guide therapy selection and assess prognosis.

Immune cells in the TME can predict resistance to PD-1 inhibitors, the backbone of immunotherapy. When PD-1 inhibitors are used, PD-1 + Tregs exhibit broader inhibition of antitumor immunity, leading to drug resistance [45]. In addition, M2-like TAMs [148] and CAFs [149] may predict drug resistance because they induce different degrees of immunosuppression. Copy number alterations have been shown to correlate with poor prognosis following PD-1 blockade therapy; substantial copy number reduction may result in the deletion of tumor suppressor genes such as PTEN [150]. The composition and abundance of gut microbiota may also predict drug resistance. Antibiotics cause dysregulation of intestinal microecology, which influences the efficacy of ICIs and can serve as a biomarker of PD-1 mAb resistance. The gut microbial composition also differs between responders and non-responders to ICIs [6]. Furthermore, some non-coding RNAs regulate the PD-1/PD-L1 axis, indicating drug resistance. Overexpression of miR200 significantly downregulates PD-L1 expression, which may reduce anti-PD therapy efficacy [151]. Anti-PD-1 resistance may also be indicated by upregulation of other immune checkpoints like TIM-3 [80]. A comprehensive analysis of biomarkers based on the various mechanisms of drug resistance may help in the selection of appropriate therapeutic options, helping to resolve drug resistance and achieve precision medicine.

Another significant problem with the increasing use of PD-1 inhibitors in clinical therapy is the development of a comprehensive biomarker system for assessing patients with a better prognosis. There are currently several predictors of PD-1 inhibitor efficacy and patient prognosis following monotherapy. PD-L1 expression [152] and tumor mutational burden (TMB) [153] are positively correlated with sensitivity to anti-PD therapy and clinical benefit. Similarly, patients with deficient mismatch repair tend to benefit from anti-PD therapy regardless of tumor type [154].

Although biomarkers are available to predict prognosis with PD-1 inhibitor monotherapy, it is not clear whether these indicators remain relevant in combination therapy. When antiangiogenic drugs and anti-PD-1 antibodies were combined, higher PD-L1 did not correlate with hypoxia or HIF-1α, implying a lack of prognostic guidance for PD-L1 expression for this combination [155]. This suggests that not all biomarkers that are meaningful for monotherapy are equally applicable to combination therapy.

In addition, biomarkers for predicting efficacy vary depending on the drug combination. When atezolizumab, an anti-PD-L1 antibody, and bevacizumab, an anti-VEGF antibody, are used in combination, high T-effector gene signature expression, and myeloid inflammation are associated with prolonged PFS [156]. For classical anti-CTLA-4 and anti-PD-1 mAb dual immunotherapy, intratumor CD4 + FOXP3-T cells expressing PD-1 is a potential biomarker, and its decline after treatment is positively correlated with better prognosis [157]. High TMB also predicts the prognosis and efficacy of combined immunotherapies. In patients with a TMB of at least 10 mutations per megabase, nivolumab plus ipilimumab had a better antitumor effect than monotherapy [130]. In addition, the quality of TMB and vaccine peptide epitopes was associated with the outcome of combined neoantigen vaccine and PD-1 blocking therapies [158]. Furthermore, the composition of the gut microbiota may be a predictor for combination therapy with TCM and anti-PD-1 mAb. Combining Gegen Qinlian decoction and anti-PD therapy enriches *Bacteroides acidifaciens* in the intestinal tract of mice; this is associated with the promotion of immunoglobulin A production and enhancement of host immunity [159].

Because of the complexity of combination therapy and associated interactions, the immune status of the TME and the host often changes, leading to the loss of function of several PD-1 inhibitor biomarkers. Therefore, it is difficult to find a widely applicable biomarker across different combination strategies Establishing a comprehensive, sensitive, accessible biomarker system will lay the foundation for diverse combination therapies.

## Discussion

Although the emergence of immunotherapy has brought hope to countless cancer patients, challenging resistance mechanisms and a lack of biomarkers still limit clinical efficacy. Many different mechanisms lead to immunotherapy resistance. Gut microbiota and host immunity are closely linked, and we further outlined the dual modulatory role of the gut microbiota and its metabolites in antitumor immunity. Epigenetic alterations can promote immune evasion and reprogram the TME. Upregulation of TIM-3, LAG-3, and VISTA activates inhibitory pathways distinct from PD-1/PD-L1, accelerating the formation of an immunosuppressive TME and mediating resistance to anti-PD therapy. The various mechanisms described above involve regulating the TME to mediate resistance to anti-PD therapy. The TME contains a diverse population of immunosuppressive cells, including TAMs, MDSCs, and Tregs, which not only restrain antitumor immunity individually, but also communicate with each other to form a complicated network. Converting the TME from protumor to antitumor is of great significance for immunotherapy. Indeed, the TME of most advanced cancers is highly heterogeneous, and despite the existence of drugs that target the TME, such as anti-CSF-1R and anti-ly6g antibodies, these drugs address resistance in only a small percentage of patients. Notably, advances in single-cell analysis have made it possible to uncover the origins, characteristics, and trajectories of critical immune cells in the TME [160]. This predicts that the main sources of resistance to anti-PD therapy can be clarified through more detailed classification.

We also discussed combined treatment strategies and synergistic treatment mechanisms. In addition to the combination therapy strategies mentioned above, many clinical trials screened for drug candidates. Recent studies have reached varying degrees of success in efficacy. For example, in a phase 1b/2 study in NK T-cell lymphoma, the anti-PD-1 antibody sintilimab in combination with the HDAC inhibitor chidamide had an expected objective response rate of 80% [161]. Similarly, a phase 2 study reported a 1-year PFS rate of 44.4% when radiotherapy plus the anti-PD-1 antibody camrelizumab were used as first-line treatment for patients with unresectable intrahepatic cholangiocarcinoma [162] (Table 1). Although multiple combination therapy strategies have addressed resistance to a certain extent, most are not approved for clinical treatment. To achieve translation to the clinic, the most critical issue is to balance efficacy with adverse effects. In addition, therapeutic timing and suitable patient populations for combination therapy need to be explored.

**Table 1:** Clinical trials of combination therapy based on anti-PD treatment

Combination	Clinical trial	Phase	α-PD-1/PD-L1		Cancer type	Primary outcome measures
TCM	NCT05735028	1	PD-1/PD-L1 inhibitor	Centipeda minima	Lung cancer	Safety, PFS, the temperature, blood pressure, complete blood count
Targeted therapy	NCT03439891	2	Nivolumab	Sorafenib	HCC	MTD, ORR
	NCT04981509	2	Atezolizumab	Bevacizumab and Erlotinib	RCC, HLRCC	Safety, ORR, DCR, PFS, OS, DOR, Response to treatment
	NCT04099836	2	Atezolizumab	Bevacizumab	NSCLC	ORR
	NCT03272217	2	Atezolizumab	Bevacizumab	UC	OS
	NCT02853331	3	Pembrolizumab	Axitinib	RCC	PFS, OS
	NCT02501096	1b/2	Pembrolizumab	Lenvatinib	Solid tumor	MTD, DLT, ORR
	NCT02454933	3	Durvalumab	Osimertinib	NSCLC	Safety
Dual immunotherapy	NCT03029780	2	Nivolumab	Ipilimumab (CTLA-4)	RCC	Safety
	NCT05004025	1	Nivolumab	Ipilimumab (CTLA-4)	Uveal melanoma	Safety, ORR
	NCT02340975	1b/2	Durvalumab	Tremelimumab (CTLA-4)	Gastric or GEJ adenocarcinoma	Safety, ORR, PFS rate
	NCT02319044	2	Durvalumab	Tremelimumab (CTLA-4)	HNSCC	ORR
	NCT02608268	1b/2	Spartalizumab	Sabatolimab (TIM-3)	Solid tumor	Safety, DLT, ORR
	NCT06003621	2	Atezolizumab	Tiragolumab(TIGIT)	Solid tumor	PFS
	NCT03743766	2	Nivolumab	Relatlimab(LAG-3)	Melanoma	Change in LAG-3 expression, PD-1 expression and tumor size, ORR
Personalized cancer vaccines	NCT04024878	1	Nivolumab	NeoVax	Ovarian cancer	Safety
	NCT03597282	1	Nivolumab	NEO-PV-01	Metastatic melanoma	Safety
HDACi	NCT03820596	1b/2	Sintilimab	Chidamide	ENKTCL	Safety, efficacy
Radiotherapy	NCT03898895	2	Camrelizumab	External radiotherapy	Unresectable iCCA	PFS

*PFS* progression-free survival, *OS* overall survival, *DOR* duration of response, *DCR* disease control rate, *MTD* maximum tolerated dose, *DLT* dose limiting toxicity, *ORR* objective response rate, *SCLC* small cell lung cancer, *HNSCC* head and neck squamous cell carcinoma, *RCC* renal cell carcinoma, *HCC* hepatocellular carcinoma, *NSCLC* non-small cell lung cancer, *UC* urothelial carcinoma, *HLRCC* hereditary leiomyomatosis and renal cell cancer, *GEJ* gastroesophageal junction, *HDACi* histone deacetylase inhibitor, *ENKTCL* extranodal natural killer/T cell lymphoma, *iCCA* intrahepatic cholangiocarcinoma

*Data from https://clinicaltrials.gov/

We also provided data for the development of a biomarker system for predicting both resistance to immunotherapy and the prognosis of patients receiving combination therapy. However, questions remain. How should the accuracy and stability of each biomarker be determined? Is there a more optimized detection method? Interestingly, a recent study reported that ICI-induced adverse effects could be predicted by changes in autoantibody profiles. For example, rash is associated with increased levels of autoantibodies, while hepatotoxicity is usually characterized by reduced levels [163]. This suggests that biomarkers can also be screened by categorizing immunotherapy-induced toxicities.

In terms of immunotherapy combination therapy strategies, developments in different fields have brought novel perspectives. Isobutyric acid, a branched short-chain fatty acid (SCFA), has potent antitumor activity and significantly reduced tumor size in mice when combined with anti-PD therapy. The function of branched SCFAs in tumor immunity has been investigated less than that of straight SCFAs, and this study may highlight the significance of the gut microbiota in immunotherapy [164]. Liujunzi extract, a TCM, modulates the PD-1/PD-L1 axis by upregulating miR-122-3p expression in HCC. Liujunzi extract activates T cells and may improve the efficacy of immunotherapy [165]. Influencing the expression of non-coding RNAs through TCM is a novel way to engage in immunotherapy. In a phase 1b/2 study, combining cadonilimab, a bispecific antibody targeting PD-1 and CTLA-4, with chemotherapy showed promising efficacy in gastroesophageal junction adenocarcinoma, with an objective response rate of 52.1% [166]. This signals that the era of bispecific antibodies is close. Certainly, efficacy is not the only concern, and the design of clinical trials for different mechanisms of drug resistance should provide an improved system for evaluating combination therapy.

In conclusion, further research into the mechanisms of immunotherapy resistance will lead to the development of novel combination therapy strategies. Establishing a standardized biomarker system will allow patients to receive more benefit from combination therapy. Continuous improvement of combination therapies will help provide breakthroughs for cancer immunotherapy.

## Data Availability

No datasets were generated or analysed during the current study.

## Abbreviations

PD-1: Programmed death 1
PD-L1: Programmed death ligand 1
ICIs: Immune checkpoint inhibitors
TCM: Traditional Chinese Medicine
CTLA-4: Cytotoxic T lymphocyte antigen 4
FDA: Food and Drug Administration
TAMs: Tumor-associated macrophages
MDSCs: Myeloid-derived suppressor cells
Tregs: Regulatory T cells
TIM-3: T-cell immunoglobulin mucin 3
LAG-3: Lymphocyte activation gene-3
VISTA: V-domain Ig suppressor of T-cell activation
VEGF: Vascular endothelial growth factor
PTEN: Phosphatase and tensin homolog
TME: Tumor microenvironment
EGFR: TKIs Epidermal growth factor receptortyrosine kinase inhibitors
HCC: Hepatocellular carcinoma
OS: Overall survival
CTLs: Cytotoxic T lymphocytes
NK: Natural killer
DCs: Dendritic cells
TGF-β: Transforming growth factor-β
IL: Interleukin
TNBC: Triple-negative breast cancer
IFN-γ: Interferon-gamma
JAK: Janus kinase
STAT3: Signaling transducer and activator of transcription 3
PI3K: Phosphoinositide 3-kinase
AKT: Protein kinase B
CSF-1R: Colony-stimulating factor-1 and its receptor
mAbs: Monoclonal antibodies
TCR: T cell receptor
G-MDSCs: Granulocytic MDSCs
ICB: Immune checkpoint blockade
MCs: Mast cells
MMP: Matrix metalloproteinase
CAFs: Cancer-associated fibroblasts
CCL: CC-chemokine ligand
CRC: Colorectal cancer
A2ARs: Adenosine 2 A receptors
HPD: Highly progressive disease
TEXs: Tumor-derived exosomes
miRNAs: MicroRNAs
Th: T helper
PFS: Progression-free survival
DNMTs: DNA methyltransferases
HDACs: Histone deacetylases
Gal-9: Galectin-9
VSIG-3: V-Set and Immunoglobulin domain containing 3
NF-κB: Nuclear factor kappa B
TILs: Tumor-infiltrating leukocytes
GDNPs: Ginseng derived nanoparticles
PZH: Pien Tze Huang
lncRNAs: Long non-coding RNAs
HOTTIP: HOXA transcript at the distal tip
PKP3: Plakophilin 3
Raf-1: Serine/threonine kinases c-Raf
VEGFR: Vascular endothelial growth factor receptor
HIF: Hypoxia-inducible factor
HGF: Hepatocyte growth factor
dMMR: Mismatch repair deficient
TMB: Tumor mutational burden
SCFA: Short chain fatty acid
